# Iminodiacetic Acid (IDA) Cation-Exchange Nonwoven Membranes for Efficient Capture of Antibodies and Antibody Fragments

**DOI:** 10.3390/membranes11070530

**Published:** 2021-07-14

**Authors:** Jinxin Fan, Cristiana Boi, Solomon Mengistu Lemma, Joseph Lavoie, Ruben G. Carbonell

**Affiliations:** 1 Department of Chemical and Biomolecular Engineering, North Carolina State University, Raleigh, NC 27695-7905, USA; jfan22@ncsu.edu (J.F.); jhlavoie@ncsu.edu (J.L.); rgcarbon@ncsu.edu (R.G.C.); 2 Golden LEAF Biomanufacturing Training and Education Center (BTEC), North Carolina State University, Raleigh, NC 27695-7905, USA; smlemma@ncsu.edu; 3 DICAM, Alma Mater Studiorum-Università di Bologna, 40131 Bologna, Italy; 4 National Institute for Innovation in Manufacturing Biopharmaceuticals (NIIMBL), Newark, DE 19711, USA

**Keywords:** membrane adsorbers, membrane chromatography, nonwoven membranes, cation-exchange, UV grafting, monoclonal antibodies (mAbs), single-chain variable fragment (scFv)

## Abstract

There is strong need to reduce the manufacturing costs and increase the downstream purification efficiency of high-value therapeutic monoclonal antibodies (mAbs). This paper explores the performance of a weak cation-exchange membrane based on the coupling of IDA to poly(butylene terephthalate) (PBT) nonwoven fabrics. Uniform and conformal layers of poly(glycidyl methacrylate) (GMA) were first grafted to the surface of the nonwovens. Then IDA was coupled to the polyGMA layers under optimized conditions, resulting in membranes with very high permeability and binding capacity. This resulted in IgG dynamic binding capacities at very short residence times (0.1–2.0 min) that are much higher than those achieved by the best cation-exchange resins. Similar results were obtained in the purification of a single-chain (scFv) antibody fragment. As is customary with membrane systems, the dynamic binding capacities did not change significantly over a wide range of residence times. Finally, the excellent separation efficiency and potential reusability of the membrane were confirmed by five consecutive cycles of mAb capture from its cell culture harvest. The present work provides significant evidence that this weak cation-exchange nonwoven fabric platform might be a suitable alternative to packed resin chromatography for low-cost, higher productivity manufacturing of therapeutic mAbs and antibody fragments.

## 1. Introduction

Therapies based on monoclonal antibodies (mAbs) and antibody fragments [1] are broadly applicable for treating chronic diseases such as cancers, rheumatoid arthritis, multiple sclerosis, and autoimmune disorders [1,2,3]. With the surging worldwide demand for these products, sales of mAb therapeutics alone are expected to rise to USD 137–200 billion in 2024 [1]. In spite of this high demand, the costs of mAb treatment place a huge burden on patients and international healthcare systems [4,5].

There are many factors leading to these high costs, including the huge expenditures and long times involved in construction, validation, and production operations. Downstream processes contribute significantly to manufacturing costs for antibodies, and the Protein A capture step is by far the most expensive. This is due in part to the high costs of the affinity resins [4], and the large number of individual procedures involved in bind-and-elute chromatographic processes (bind, wash, elute, regenerate). Diffusional limitations in the resin make column chromatography an inherently slow process [6,7], whether it is used for product capture or product polishing to remove impurities. Cumbersome column sanitation and validation processes after each purification cycle greatly increase the production costs due to buffer usage, time, and labor [8,9]. From a broader perspective, the explosion in new product modalities, including bispecific antibodies, antibody–drug conjugates, and single-domain antibodies [3], has increased industrial demands for flexible, single-use, high-capacity, high-throughput processes that are easily adaptable to a wide range of biologics.

As a result of these factors, several alternative, and potentially more efficient separation approaches for mAb purification are being considered [9,10,11,12,13,14]. Membrane chromatography is widely regarded as a promising alternative to resin chromatography [15,16]. The relatively high permeability and lack of diffusional resistances for product adsorption result in low pressure drops and shorter residence times [5]. In addition, membranes lend themselves to single-use, modular operations at a variety of process scales, and are an excellent fit for continuous downstream processing in next-generation biomanufacturing [17,18]. Chromatographic membranes have been successfully implemented into polishing processes in mAb manufacturing, particularly for the removal of impurities in flow-through mode [6]. However, the use of membrane chromatography for product capture has lagged behind because of the low binding capacity of cast membranes resulting from their low available specific surface areas for binding [7,19].

In recent years, several breakthroughs have been made in the development of membrane structures with increased binding capacities. Several groups have reported the development of electrospun nanofibrous membranes with improved porosity and specific surface area and high ion-exchange protein-binding capacities [5,20,21]. Rajesh et al. coupled cationic polyacids onto self-supported cellulose nanofibrous structures, and the resultant membranes exhibited a binding capacity of 508 mg/g for lysozyme with very short residence time [15]. Fu et al. used an ethylene–vinyl nanofibrous membrane as the base matrix for direct reaction with citric acid (as cation-exchange ligand), and the prepared membrane showed a lysozyme binding capacity of 250 mg/g [20]. On the other hand, constructing a 3D layer on the membrane surface by various grafting methods has also been demonstrated as an effective way to increase the protein-binding capacity [22,23,24]. Husson et al. used an atom transfer radical polymerization (ATRP) method for fabricating various ion-exchange membranes exhibiting high productivity for protein purification [25,26]. Sahadevan et al. developed an anion-exchange membrane through redox polymerization, and illustrated its potential use in virus removal during downstream processing [24]. Other groups have demonstrated the use of high energy beams and light treatment on the membrane surface [27,28,29,30]. Ulbricht et al. demonstrated that directly grafting 2-trimethylammonioethyl methacrylate with photo-initiated graft copolymerization is an effective way to obtain anion-exchange membranes with a high bovine serum albumin (BSA)-binding capacity of 80 mg/mL [31]. Saito et al. applied radiation-induced graft polymerization to form multi-layer sulfonic acid groups on porous hollow-fiber membranes and obtained a binding capacity for lysozyme of 130 mg/g [29]. UV grafting is also a reliable and robust approach for fabricating high performance membranes at relatively low costs [27,28].

Since commercial chromatographic membranes are reported to cost much more than counterpart resins on a per volume basis, greatly limiting their use as single use disposables for bioseparations [25,32], inexpensive nonwoven membranes such as polypropylene and poly(butylene terephthalate) (PBT) were successfully modified in our lab by UV grafting. The results were membranes that exhibited high protein-binding capacity and a significant potential to realize single-use membrane capture chromatography [28,33]. In our previous work, a strong cation-exchange (CEX) membrane with sulfonic acid as ligand (CEX-SO_3_ membrane) was fabricated based on a PBT nonwoven and exhibited an excellent protein-binding capacity of 712.9 mg human polyclonal immunoglobulin G (hIgG)/per g of membrane at equilibrium [34]. It is well known that the sulfonate group is very stable and completely ionized over a wide pH range, while the carboxyl group commonly has weak cation-exchange ligands with variable ionization depending on the solution pH, and this can generate selectivity results that are different from those of strong cation-exchange ligands [35,36]. These two modes are usually employed for screening separation conditions during process development [36]. In addition, attaching carboxyl groups on the membrane has resulted in improved membrane hydrophilicity and permeability [37,38,39]. For instance, Yuan et al. found that the water flux of a graphene oxide nanofiltration membrane was increased by about 20% after carboxylation through coupling glycine onto the membrane [37].

In the current study, we developed a weak cation-exchange membrane by attaching iminodiacetic acid (IDA) onto a PBT nonwoven membrane that was modified by UV grating of glycidyl methacrylate (GMA). We expected that the IDA, which harbors two charged carboxyl groups, could endow the membrane with high protein-binding capacity while enhancing the hydrophilicity of the polyGMA grafted membrane and result in excellent flow permeability. During membrane preparation, the influence of IDA coupling pH, temperature, and time were investigated, and the prepared membranes were compared to commercial resins as well as to CEX-SO_3_ strong cation-exchange membranes. The protein-binding capacity of the prepared CEX-IDA membrane was evaluated with spiked polyclonal human IgG and a single-chain antibody fragment (scFv) in buffer, or in CHO cell supernatant. Membrane selectivity was investigated in the separation of IgG from a mixture of IgG with bovine serum albumin (BSA). Finally, the evaluation of five reuse cycles for capturing an mAb from a cell culture fluid was carried out. This work illustrates the potential of high-capacity, weak cation-exchange membranes to meet the current demand for novel product capture steps for mAbs production.

## 2. Experimental

### 2.1. Materials

PBT nonwoven membranes with base weight 52 g/m^2^, fiber diameter 3 μm, thickness 300 μm, specific surface area 0.72 ± 0.10 m^2^/g, and mean pore size 8.0 ± 0.5 μm, were kindly supplied by Macopharma (Tourcoing, France). GMA was purchased from Reagent World (Ontario, CA, USA). Bovine serum albumin (BSA), benzophenone (BP) and Toluidine Blue O (TBO) were obtained from Sigma-Aldrich (St. Louis, MO, USA). Methanol, butanol, tetrahydrofuran (THF), isopropyl alcohol (IPA), and iminodiacetic acid (IDA) were purchased from Fisher Scientific (Fairlawn, NJ, USA). All chemicals used for buffer preparation were of analytical grade. Human polyclonal IgG was purchased from Athens Research & Technology, Inc. (Athens, GA, USA). Sodium dodecyl sulfate polyacrylamide gel electrophoresis (SDS-PAGE) kits were from Bio-Rad (Hercules, CA, USA), the CHO HCP ELISA Kit (F550) was purchased from Cygnus (Southport, NC, USA), and the Quant-iT™ PicoGreen™ dsDNA Assay Kit was bought from Fisher Scientific (Fairlawn, NJ, USA). The above kits were used according to the manufacturers’ instructions. The CHO supernatant was provided by the Biomanufacturing Training and Education Center (BTEC) of North Carolina State University (Raleigh, NC, USA).

### 2.2. Preparation of Cation-Exchange Membranes

PolyGMA was grafted onto the PBT nonwoven membrane by UV treatment. The grafting solution was prepared by dissolving BP in a GMA monomer solution (a molar ratio of 1:20) with butanol as solvent (20%, *v*/*v*). The PBT nonwoven membrane (75 × 50 mm) was sprayed with 1.0 mL GMA grafting solution, then sandwiched between two glass slides and exposed to UV light using a lamp (model EN-180, Spectronics Corporation, Westbury, NY, USA) for grafting GMA (wavelength: 365 nm, intensity: 5 mW/cm^2^, distance: 3 mm). Finally, the polyGMA modified membrane was washed with THF and methanol under sonication, followed by drying overnight at room temperature. The degree of GMA grafting was based on the polyGMA grafted weight gain (WG) which was calculated as the increased weight after grafting divided by the original membrane weight [33]. The 15% and 20% WG polyGMA grafted membranes were obtained by grafting GMA for 17 and 22 min. The polyGMA activated membrane was then soaked in an IDA solution (66.2 mg/mL in 20% IPA/water (*v*/*v*)) for ligand coupling. The 15% WG polyGMA grafted membranes with 7 h coupling time at 60 °C were used to investigate the impact of pH (8.5–13) on IDA coupling. Under the optimized pH, the CEX membranes prepared by coupling IDA onto the 20% WG polyGMA grafted membrane at 60 °C for 16 h, or 80 °C for 7 h were studied in the subsequent experiments. The unreacted epoxy groups were hydrolyzed with 0.1 M sulfuric acid at 50 °C (16 h) for reducing non-specific protein binding. The functionalized CEX-IDA membranes were washed three times with pure water and dried overnight at room temperature.

### 2.3. Membrane Characterization

The chemical composition of the membrane surface was measured by attenuated total reflection Fourier-transform infrared (ATR-FTIR) spectra (from 500 to 4000 cm^−1^) using a Nicolet spectrometer (Thermo-Nicolet 6700, Thermo Fisher Scientific, Pittsburgh, PA, USA) with 64 scans at a resolution of 4 cm^−1^. A scanning electron microscope (SEM, Hitachi S-3200 N, Hitachi High-Tech, Schaumburg, IL, USA) was used to observe the morphology of the membrane surface (accelerating voltage: 5.0 kV). The density of the carboxyl groups on the membrane was measured with Toluidine Blue O (TBO) assay according to the method reported by Sano et al. [40]. The membrane samples (10 × 10 mm) were immersed in 1.5 mL 0.5 mM TBO solution (pH 10) for 3 h, and then washed thoroughly with water (adjusted to pH 10). The subsequent TBO desorption was conducted at 50% (*v*/*v*) acetic acid solution. TBO adsorption, assuming that TBO and carboxyl were complexed at a molar ratio of 1:1, was measured at 633 nm with UV-vis spectrophotometer (Agilent Technologies, G1103A, Santa Clara, CA, USA). Water uptake of the nonwoven membrane was evaluated with a dynamic contact angle analyzer (DCA-315, Thermo Fisher Scientific, Pittsburgh, PA, USA). The membrane samples (15 × 10 mm) were hung on the hook and slowly immersed into water. The weight of water uptake was recorded instantly once the membrane touched the water surface. The amount of IDA immobilized on the membrane was determined through nitrogen content analysis with a PE 2400 CHN elemental analyzer (PerkinElmer Inc., Waltham, MA, USA) by combusting samples to elemental N_2_ gases and then detecting N_2_.

### 2.4. Static Protein-Binding Experiments

The prepared membrane samples (~15 mg, 15% or 20% WG) were soaked in 3 mL of IgG solution at 10 mg/mL in 50 mM acetate buffer at pH 5.5 (equilibration buffer) at room temperature for 16 h. The membranes were subsequently washed with equilibration buffer for five times, followed with an elution step where the IgG was recovered. The membranes were incubated in 3 mL elution buffer (equilibration buffer with additional 1 M NaCl) for 2 h, after incubation, the IgG concentration was measured by absorbance readings at 280 nm. The static binding capacity (SBC) of the membranes was calculated by dividing the eluted IgG mass by the membrane weight.

### 2.5. Dynamic Protein-Binding Experiments

A stack of 12 membrane layers of 25 mm diameter was placed on an Omnifit column (Diba Industries, Inc., Cambridge, UK) holder (diameter 25 mm) and was connected to a FPLC system AKTA pure (GE Healthcare Bioscience, Uppsala, Sweden). The chromatographic system monitored and recorded process parameters including UV absorbance, pH, conductivity, and back pressure. First, 50 mL of equilibration buffer (50 mM acetate buffer, pH 5.5) was used to equilibrate the membranes at 1.0 mL/min, then 15 mL feed (10 mg IgG/mL in equilibration buffer) was injected (IgG loading of 100 mg per mL membrane volume). The unbound protein was washed with equilibration buffer until the UV absorbance returned to zero. Finally, the bound protein was stripped by elution buffer (equilibration buffer with 1 M NaCl addition) at 1.0 mL/min. The mass of eluted IgG was determined by UV-vis spectrophotometer at 280 nm with a calibration curve based on known concentrations of IgG. The dynamic binding capacity (DBC) of the membrane was calculated as the mass of IgG eluted divided by the volume of the membrane bed. Various flow rates (0.33–16.6 mL/min) corresponding to 5.0–0.1 min residence time (RT) for sample loading were applied to study their influence on the DBC.

### 2.6. DBC_100%_ Measurement by Capturing Spiked IgG and scFv from CHO Supernatants

CHO supernatant was first diafiltered with equilibration buffer (50 mM pH 5.5 acetate), then IgG powder or scFv concentrated solution were, separately, spiked into this solution with a final concentration of 2.0 mg/mL. A 0.24 mL membrane bed (10 mm diameter) and an IgG or scFv loading of 200 mg per mL membrane volume (a sufficient protein loading to reach maximum binding capacity) were applied. Three different RTs (0.1, 0.5 and 2.0 min) were used during protein loading while equilibration, wash, and elution processes were conducted at 1.0 min RT. The eluted IgG and scFv were quantified with UV spectrophotometer at 280 nm. The binding capacity was calculated as the mass of the eluted IgG or scFv divided by the membrane volume. The HCP and DNA content were determined using a CHO HCP ELISA Kit (F550) and a Quant-iT™ PicoGreen™ dsDNA Assay Kit, respectively. The log reduction values (LRV) of HCP and DNA clearance were calculated by log_10_ ratio of HCP or DNA in the feed solution to the elution fractions.

### 2.7. Selectivity Evaluation by Separation of IgG from IgG/BSA Mixtures

The separation of IgG from a solution containing 1.0 mg/mL IgG and 4.0 mg/mL BSA (similar to the IgG/human serum albumin ratio in human plasma) was conducted using a column with 12 circular coupons of membrane (diameter: 10 mm, volume: 0.242 mL) at 1.0 min RT. 5.0 mL of equilibration buffer (20 mM phosphate buffer, pH 6.5) was pumped through the column for equilibration, followed by injection of 1.0 mL feed solution. The membrane bed was then washed with equilibration buffer until the UV absorbance reached baseline. Finally, 3.0 mL elution buffer (equilibration buffer with 1 M NaCl addition) was applied to elute the bound proteins. The flow-through fraction and elution fraction were collected and analyzed by SDS-PAGE.

### 2.8. Capturing Monoclonal Antibody from Cell Culture Supernatant with Five Reuse Cycles

The cell culture supernatant was adjusted to pH 5.5 and conductivity ~3.0 mS/cm by dialysis with equilibration buffer (50 mM acetate buffer, pH 5.5). After membrane equilibration with 8 mL equilibration buffer, 14 mL feed solution was loaded onto a 0.265 mL membrane bed (loading 86.4 mg mAb per mL membrane volume). The membrane bed was then washed with binding buffer till the UV absorbance returned to baseline. The bound mAb was collected by applying the elution buffer, followed by flushing the membrane with 5.3 mL equilibration buffer. Five repeated cycles were carried out at 1.0 min RT for reusability evaluation. The mAb concentration and purity were measured with a protein G column (GE healthcare): 5 mL equilibration buffer (20 mM phosphate buffered saline, pH 7.4) was fed at 1.0 mL/min, then 0.1 mL sample solution was injected; after column wash with 10 mL equilibration buffer, a 0.1 M glycine buffer (pH 2.8) was used to elute the bound IgG at 1.0 mL/min. The recovery of mAb was calculated as the mass ratio of IgG in the elution fraction to that in the applied feed. The purity of IgG was the peak area ratio of the eluted IgG to the total protein.

## 3. Results and Discussion

### 3.1. Preparation and Characterization of CEX-IDA Membranes

**Figure 1:**
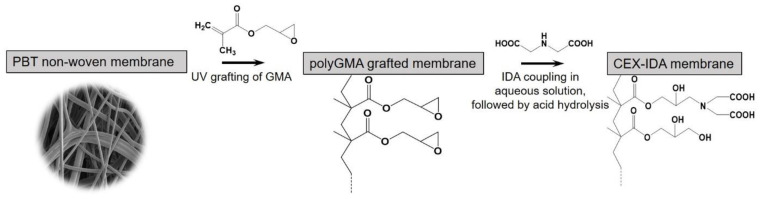
Schematic diagram of CEX-IDA membrane preparation.

Figure 1 shows the preparation procedure used to develop the cation-exchange nonwoven membranes. The UV grafting of GMA resulted in a uniform, conformal coating around each individual PBT fiber, providing available epoxy groups for further functionalization, and an increased volume for subsequent protein binding by ion exchange. The IDA was covalently coupled to the GMA by reaction of the imine with the epoxy groups in GMA in aqueous solution. To reduce non-specific adsorption, the unreacted epoxy groups were hydrolyzed in acidic solution after IDA coupling. It has been reported that the solution pH can have a strong effect on the coupling efficiency of amines with other functional groups [32]. As a result, we investigated the effect of pH on the resulting IDA ligand density and the effect this has on IgG binding capacity under static conditions using a polyGMA grafted nonwoven fabric with a 15% WG at a fixed temperature and reaction time.

**Figure 2:**
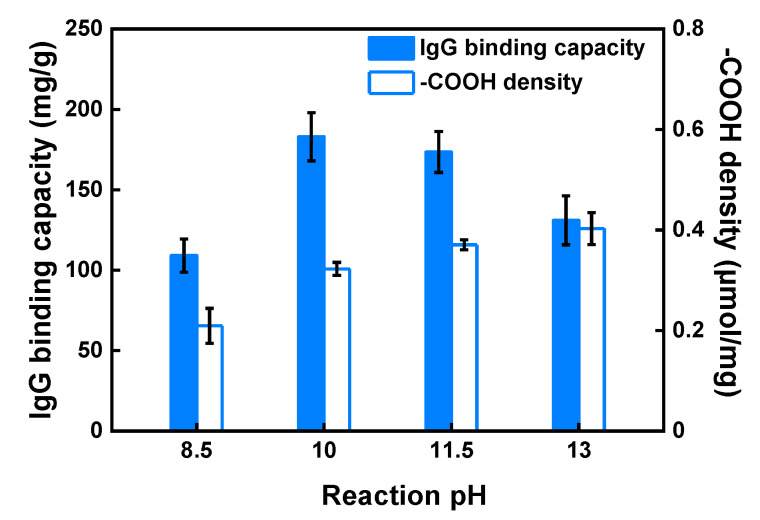
Influence of reaction pH for IDA coupling on IgG static binding capacity and -COOH density on CEX-IDA membranes. Preparation conditions: polyGMA grafted at 15% WG, IDA coupling for 7 h at 60 °C.

As seen in Figure 2, the density of grafted carboxyl groups increased monotonically with pH in the range of pH 8.5 to 13.0. This resulted in an increase in IgG static binding capacity in the pH range of 8.5 to 10.0. However, the binding capacity declined as the pH was increased further from 10.0 to 13.0, even though the ligand density increased in that range. This can be ascribed to a limited accessibility for IgG to crowded ligand binding sites in the grafted layers. A similar observation was also reported by Wrzosek et al. in a study of the influence of resin ligand density on IgG binding capacity. They explained that this phenomenon was due to increased steric hindrance and narrowed pores sizes at increased ligand density [41]. Due to the higher binding capacity obtained by linking IDA onto the membranes at pH 10.0, we employed this reaction condition for the studies that followed. Prior work in our lab [34,42] indicated that ion-exchange membranes prepared by grafting polyGMA at 20% WG showed higher protein-binding capacity while maintaining excellent flow permeability. As a result, all CEX-IDA membranes used in the following studies were prepared based on 20% WG polyGMA grafted membranes.

**Figure 3:**
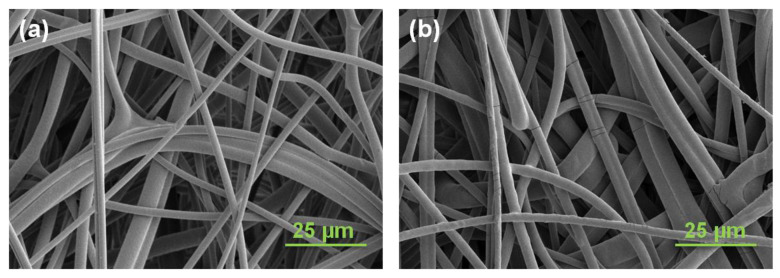
SEM images of the membrane surface for (**a**) pristine PBT membrane and (**b**) final CEX membrane. Preparation conditions: polyGMA grafted at 20% WG, IDA coupling for 16 h at 60 °C.

ATR-FTIR is an effective method to characterize the surface chemical structure of modified membranes. The spectrum of the unmodified PBT membrane was compared to the polyGMA grafted membrane. As evident in Figure S1, the appearance of the epoxy peak at 905 cm^−1^ in the polyGMA grafted membrane sample confirmed the effective grafting by UV radiation. As expected, the epoxy peak disappeared after IDA coupling, while the strong C-N stretching peak at 1160 cm^−1^ and a broad weak O-H stretching peak around 3100–3400 cm^−1^ emerged, indicating the successful immobilization of IDA onto the membrane. Since the membrane structure plays a critical role in solute transport within the inner pores, the membrane morphology was studied by SEM. As shown in Figure 3a, the unmodified PBT nonwoven membrane is composed of randomly intersecting fibers which form a tortuous porous structure. After membrane functionalization, the network of fibers became denser as evidenced by the increased opaqueness of the membranes. Nevertheless, the open and interconnected porous structure was preserved, ensuring the accessibility and flexibility of interactions between proteins and adsorptive ligands (Figure 3b).

**Figure 4:**
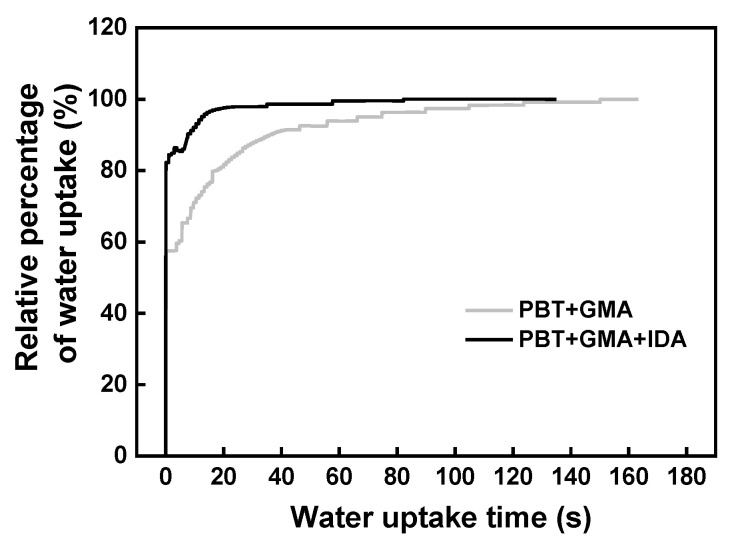
Relative percentage of water uptake (mass of water adsorbed at a given time divided by the maximum mass of water uptake) on the membrane as a function of time. The polyGMA grafted membrane: PBT+GMA and CEX-IDA membrane: PBT+GMA+IDA. Preparation conditions: polyGMA grafted at 20% WG, IDA coupling for 16 h at 60 °C.

To evaluate wettability and permeability of the membranes, water uptake measurements were carried out and the results were plotted as a function of time as shown in Figure 4. As expected, there was no water uptake for the original membrane due to the hydrophobic surface of PBT (water contact angle 138°). Once polyGMA was grafted, the water drop began to penetrate the membrane, suggesting that the hydrophilicity improved with the introduction of epoxy groups, and around 100 s was needed to reach uptake saturation. The water saturation time was further reduced to 20 s for the IDA modified membrane, indicating that IDA with its abundant COOH groups further improved membrane hydrophilicity. This property tends to decrease non-specific protein adsorption and ensure satisfactory water flow.

Additional pressure drop-flow rate experiments showed that the resultant flow permeability coefficient calculated by Darcy’s law was approximately 1.0 × 10^−8^ cm^2^ [43] (shown in Figure S2). This is somewhat higher than the flow permeability (8.3 × 10^−9^ cm^2^) reported for hydroxylated (poly) methacrylate-based resin with particle diameters in the range of 60–90 µm [44]. This confirms the great advantage of the well-opened porous structure and the high hydrophilicity of this cation-exchange membrane.

### 3.2. DBC Evaluation and Comparison

**Figure 5:**
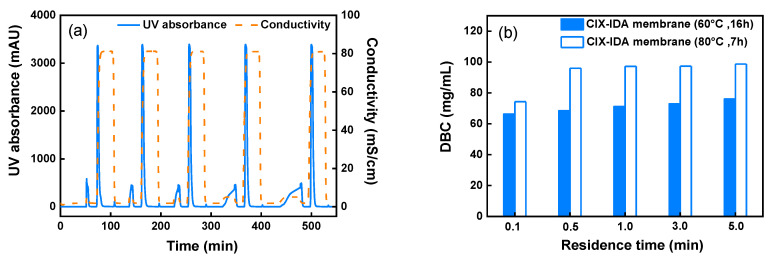
Results of five consecutive IgG bind-and-elute cycles at different RTs from 0.1 to 5.0 min. (**a**) Chromatograms for CEX-IDA membranes prepared by coupling IDA at 80 °C, 12 layers and (**b**) DBC comparison for membranes prepared at different IDA coupling conditions.

Dynamic flow experiments provide useful insights into the performance of the novel CEX-IDA membranes in real applications. Initially, the membranes were employed to evaluate the effect of operating flowrate on the dynamic binding capacity (DBC) for polyclonal IgG in non-competitive conditions at five different flow rates (~0.33–16.6 mL/min) corresponding to residence times (RT) in the range of 5.0–0.1 min. The operating pH was set at 5.5, where the positively charged IgG could be captured by negatively charged IDA ligands. The bound protein was recovered by a high-conductivity buffer that screened the charges on both IgG and ligands. Figure 5a presents the chromatograms of five consecutive bind-and-elute cycles of CEX-IDA membrane prepared by 7 h coupling IDA at 80 °C. The flow-through peak span increased at longer residence times, while the elution peak areas remained almost the same. The DBC at 0.1 min RT was 74.3 mg/mL, which was lower than those obtained at RTs from 0.5 to 5.0 min where the DBC was constant around 96.0–98.7 mg/mL. In the range of RT of 0.5 min and above, the membrane showed no diffusional limitations, as normally observed with other membrane adsorbers [45]. At the shortest RT of 0.1 min, there is an apparent diffusional limitation for the transport of proteins into the grafted layers [30]. The DBC values were compared to those measured with CEX-IDA membranes prepared at 60 °C and 16 h IDA coupling time (Figure 5b and Figure S3). The former showed a high binding capacity at all the RTs investigated. While the pressure drop per unit bed height during protein sample loading was also found to be higher for membranes prepared at 80 °C (11.8 kPa/cm vs. 8.0 kPa/cm at 1.0 min RT), this could be ascribed to the higher ligand density, 5.5 vs. 5.0 μmol (IDA/mg), leading to a stronger binding ability. On the other hand, the higher ligand density also promoted pore narrowing by extension of grafted polymer with electrostatic repulsion in low-ionic-strength protein loading buffer (decreasing membrane permeability).

Due to its higher DBC, the following studies were carried out only with CEX-IDA membrane prepared by 7 h coupling of IDA at 80 °C. Compared to the strong cation-exchange membrane CEX-SO_3_ prepared in our lab using the same nonwoven polymer matrix, the CEX-IDA membrane had a slightly lower DBC (96.0 mg/mL vs. 99.2 mg/mL) at 0.5 min RT as reported in Table 1. This is consistent with the SBC comparison (605.2 mg/g vs. 712.9 mg/g) resulting in part from the lower ligand density for IDA (5.5 µmol/mg vs. 5.8 µmol/mg). While, the CEX-IDA membrane also exhibited a slightly lower pressure drop conducted with equilibration buffer, as it was less swelled because of the fewer charged ligands on the membrane.

To evaluate this novel CEX-IDA membrane at conditions typically employed in the biomanufacturing industry, the DBC at 10% breakthrough, the dynamic binding capacity measured at 10% breakthrough (DBC_10%_) was determined. At 0.1 min RT, the DBC_10%_ was 76.6 mg/mL, while a higher binding capacity of 108.5 mg/mL was obtained at 1.0 min RT (Figures S5 and S6). With a minimal loss of IgG in the flow-through and washing steps, a high recovery of 98.9% and 94.2% could be obtained at 0.1 min and 1.0 min RT, respectively. When compared with other commercial cation-exchange membranes, the DBC_10%_ was much higher than that of Sartobind S membrane (~20 mg IgG/mL around 0.15 min RT) and Mustang S membrane (~22 mg IgG/mL around 2.85 min RT) [46], and comparable to that of Natrix HD-C membrane (74 mg/mL at 0.1 min RT) [9]. Moreover, at short residence times, the achieved binding capacity was even higher than that of commercial Capto S resins (55 mg IgG/mL at 0.5 min RT and ~90 mg IgG/mL at 1.0 min RT) [47]. Considering that the CEX-IDA membranes were designed for fast capture of target proteins, they are indeed a powerful tool for improving the productivity of protein purification.

### 3.3. Evaluation of CEX-IDA Membranes in Competitive Conditions

**Figure 6:**
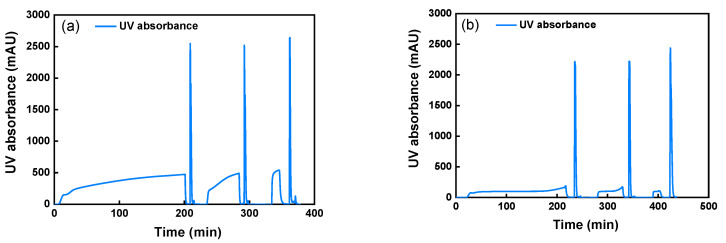
Chromatograms of bind-and-elute cycles at 0.1 min, 0.5 min, and 2.0 min RT, using 12 membrane layers of 10 mm diameter. After running at 0.5 min residence time, the membrane bed was replaced by a new one. Experiments performed to capture: (**a**) spiked IgG and (**b**) scFv from CHO culture supernatant.

The impurities present in cell culture supernatants can interfere with target protein binding onto the adsorbent [48]. To understand the extent of this phenomena on CEX-IDA membranes, we evaluated the membrane dynamic binding capacity (DBC_100%_) for spiked human polyclonal IgG and scFv from a CHO supernatant in separate experiments. The resulting chromatograms at three short residence times from 0.1 min to 2.0 min are shown in Figure 6, and the quantitative results of the analysis are displayed in Table 2. With a protein loading of 200 mg per mL of membrane volume, the DBC_100%_ improved as the RT increased from 0.1 to 0.5 min for both proteins, and further increased slightly by 2–3 mg/mL at a higher RT of 2.0 min. This trend confirms the results obtained in non-competitive experiments with pure IgG in buffer, indicating that the lower binding capacity at shorter RT is mainly caused by diffusion limitations rather than the interference of the impurities in the supernatant. In comparison with IgG binding, the higher mass binding capacity obtained for scFv (Table 2) can be explained by its smaller molecule weight (25 kDa vs. 150 kDa for IgG). As seen in Table 2, a significant fraction of the host cell proteins (HCP) and DNA contaminants were removed during IgG and scFv capture steps. The best contaminant removal performance was found with the scFv spiked solution compared to the polyclonal human IgG spiked solution. This phenomenon may be attributed to a potentially stronger interaction between scFv and cation-exchange ligands, as well as to the easier accessibility for smaller scFv proteins. Indeed, scFv occupied more binding sites on the membrane, resulting in a decreased HCP and DNA binding. It is well known that protein A columns are unable to bind scFv protein with no Fc domain [49,50]. On the other hand, protein L affinity purification designed for specific scFv capture is usually too costly to implement in large-scale production. Thus, cation-exchange-based product capture can play an important role in scFv production and in this context the CEX-IDA membrane offers a potentially viable alternative for preliminary purification of scFv with a very large protein-binding capacity.

### 3.4. Membrane Selectivity Evaluation

**Figure 7:**
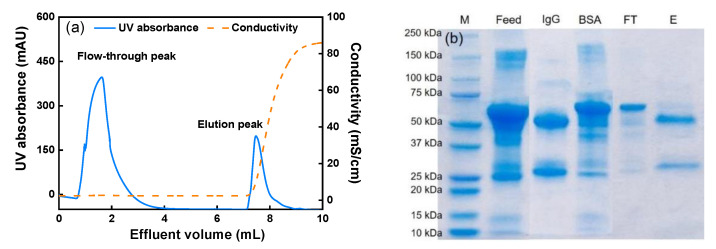
(**a**) The chromatogram of IgG separation from IgG/BSA mixture at 0.24 mL/min flow rate using 12 membrane layers with 10 mm diameter (0.24 mL membrane volume) and (**b**) the corresponding SDS-PAGE. Lane M: molecular weight markers, feed: IgG/BSA mixture, IgG: IgG standard, BSA: BSA standard, FT: flow-through fraction, E: elution fraction.

To investigate the selectivity of the prepared membranes, a protein mixture containing polyclonal human IgG and BSA (mass ratio: 1:4) was loaded onto the membrane bed at 1.0 min RT. As the separation pH was set at 6.5, the positively charged IgG (pI~8.2) as the target protein should be retained on the membrane. On the other hand, BSA (pI = 4.7) as the impurity should not bind to the membrane and can be collected in the flow-through as both BSA and the membrane were negatively charged. As depicted in Figure 7a, the two peaks in the chromatogram represent the flow-through and the elution fraction (generated with a high-conductivity solution). The collected fractions were analyzed with SDS-PAGE and the results are shown in Figure 7b. In the elution lane, the IgG bands containing the light chains (~25 kDa) and heavy chains (50 kDa) of the polyclonal human IgG are clearly displayed, indicating that IgG was captured by the CEX-IDA membrane during loading. Meanwhile, the majority of BSA (~67 kDa) is only present in the flow-through fraction. As a result, a high IgG purity of 95% in the elution fraction was achieved. Therefore, the successful fractionation of IgG from an IgG/BSA mixture confirms the selectivity of CEX-IDA membranes, which is advantageous for treating IgG contained streams.

### 3.5. Capture of a Monoclonal Antibody from Cell Culture Supernatant

**Figure 8:**
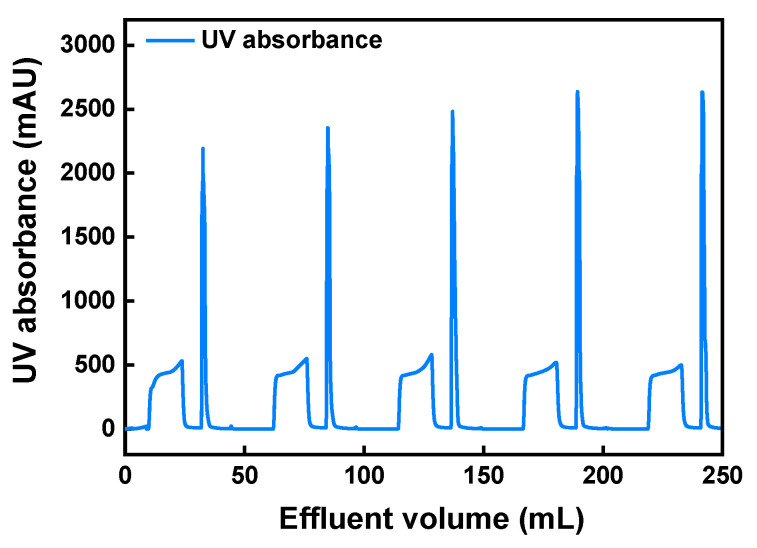
Chromatogram of five consecutive cycles of mAb capture from cell culture fluid with 12 layers of CEX-IDA membrane with 10 mm diameter (0.265 mL membrane volume) at 1 min RT (0.265 mL/min flow rate).

As robust reusability of single use membrane is highly desired for conducting many chromatographic cycles in one campaign [51], the stability of membrane performance in five cycles of product capture and elution of an expressed mAb from its cell culture fluid was studied. Before chromatography, the supernatant was dialyzed with equilibration buffer to reduce the conductivity of the solution. An mAb loading of 86.4 mg per mL of membrane volume was applied for each cycle. Figure 8 shows the chromatogram of five consecutive cycles at 1.0 min RT. According to Table 3, the mAb recoveries during the five cycles ranged from 94.2 to 99.5%, with a stable binding capacity of 81.8–86.4 mg/mL. Given that the commercial protein A resins have a binding capacity of 40–60 mg/mL operated at 4–6 min RT [4,49], the prepared membrane is promising for reducing the number of chromatographic cycles and improving the process throughput. A variety of cation-exchange resins were also evaluated for monoclonal antibody capture by other research groups. For instance, Tao et al. reported an mAb binding capacity around 65–70 mg/mL at 6.0 min RT under a relatively high conductivity of 12–15 mS/cm [50]. It could be reasonable to anticipate that the binding capacity of the CEX-IDA membrane would decrease due to charge shielding when using a high conductivity solution. This impact could be alleviated by adjusting to a lower pH for maintaining the electrostatic attraction between protein and ligands. Regarding other cation-exchange membranes tested for mAb capture, the Natrix HD-C membrane was also demonstrated to be an efficient tool for preliminary purification with a high binding capacity of 49.5 mg/mL at 0.1 min RT [9]. Under this short residence time, the newly developed CEX-IDA membrane is very likely to obtain a higher binding capacity for mAb capture considering that DBC_10%_ at 0.1 RT min was 76.6 mg/mL.

The purity of mAb shown in Table 3 was improved from 43.9% in the feed solution to more than 87% in the eluate (shown in Figure S7). The consistent purities (87.2–87.9%) obtained from five cycles (Figure S8) confirmed the robust separation performance of the CEX-IDA membrane. The obtained purities are lower than those obtained by commercial protein A resins (normally more than 98%) due to a high specificity between protein A and the Fc region on mAbs [49], while the obtained purities in this study are similar to those reported using cation-exchange resins [52]. The impurity clearance of this capture step was 1.0–1.2 LRV for DNA and 0.3–0.4 LRV for HCP (some basic HCPs were co-eluted). When the HCP clearance was defined as the ratio of total HCP mass in the load to that in the elution fraction, the values were around 2.4–2.9, which was similar to that (~2) obtained when using a Gigacap CM column (with the same ligand: carboxyl group) for mAb capture [50]. HCP clearance performance of a cation-exchange based separation is generally related to the loading pH, loading conductivity and salt gradient in the elution process as well as the charge characteristics of mAbs [50,53,54]. These separation conditions could be optimized in future work for improving HCP removal. In addition, protein aggregates were reduced from 3.5% in the feed solution to 1.1–1.5% in the elution fraction. With the recommendable separation performance and excellent reusability in the five reuse cycles for mAb purification, the prepared CEX-IDA membrane offers an effective alternative for mAb capture from CHO cell culture supernatants.

## 4. Conclusions

To address the issue of the high production costs in mAb production, a simple and effective combination of inexpensive materials and low-cost preparation methods was used for the development of high performance, potentially single-use disposable, weak cation-exchange membranes for product capture, using IDA as ligand. UV grafting of GMA was used to activate the PBT fabric and create grafted layers around each fiber for increased protein binding. Subsequent IDA coupling imparted the membrane with a high cation-exchange ability. The DBC (96.0–98.7 mg/mL) of the prepared membranes for polyclonal human IgG was largely independent of residence times (RT) from 0.5 to 5.0 min, though it decreased to 74.3 mg/mL at 0.1 min RT due to diffusional limitations in the grafted layers. The DBC obtained at 0.5 min RT was slightly lower than that of CEX-SO_3_ strong cation-exchange membranes due to the lower ligand density, although the CEX-IDA membrane exhibited a better permeability in equilibration buffer. The achieved high DBC_10%_ of 76.6 mg/mL at 0.1 min RT indicates a very high productivity for IgG capture. The observed DBC_100%_ for capturing polyclonal human IgG and scFv from mammalian cell culture were fairly constant for RTs in the range of 0.1–2.0 min. Noticeably, the CEX-IDA membrane exhibited a very high scFv binding capacity of ~150 mg/mL and provides a valuable alternative for product capture steps during the production of mAb fragment products. When purifying IgG from a mixture containing BSA as impurity protein for selectivity evaluation, a high IgG purity of 95% could be obtained. Finally, the prepared CEX-IDA membrane could be reused for five bind–elute cycles in capturing mAbs from cell culture harvest at 1.0 min RT with high recovery of 94.2–99.5%. The obtained purity was 87.2–87.9% as some basic impurities were co-eluted, but these values are comparable to those obtained using cation-exchange resins. The achieved high mAb loading capacity of 86.4 mg per mL of membrane volume at 1.0 min RT could be highly beneficial for high-throughput production. The present work provides further indication that cation-exchange nonwoven membranes offer highly viable alternatives to chromatographic resins as efficient, high-productivity product capture steps in the production of high-value biotherapeutics.

## Figures and Tables

**Table 1:** Comparison of CEX-IDA and CEX-SO_3_ membranes ^1^ prepared by coupling of ligands for 7 h at 80 °C.

Nonwoven Membrane	Ligand Density (µmol/mg)	DBC at 0.5 min RT (mg/mL)	SBC (mg/g)	Pressure Drop (kPa/cm) at 146.7 cm/h
CEX-IDA	5.5	96.0	605.2 (Figure S4)	82.5
CEX-SO_3_	5.8	99.2	712.9	87.2

^1^ For the two membrane types, the experiments were performed using the same conditions with 12 membrane layers packed in a 12 cm diameter column. All details of CEX-SO_3_ membrane experiments are reported in [34].

**Table 2:** Performance of capturing spiked IgG and scFv from CHO culture supernatant (protein loading: 200 mg/mL membrane volume; membrane volume: 0.24 mL; flow rate: 0.12–2.4 mL/min).

Protein Used	RT (min)	DBC_100%_ (mg/mL)	DBC_100%_ (µmol/mL)	HCP (LRV)	DNA (LRV)
IgG	0.1	127.5	0.85	0.8	0.7
IgG	0.5	136.5	0.91	1.0	0.9
IgG	2.0	139.6	0.93	0.9	0.7
scFv	0.1	143.2	5.7	1.2	1.2
scFv	0.5	151.4	6.1	1.0	1.0
scFv	2.0	154.5	6.2	1.2	1.0

**Table 3:** Results of five consecutive cycles for mAb capture from cell culture fluid.

Purification Cycle	DBC ^1^ (mg/mL)	Recovery (%)	Purity (%)	HCP (LRV)	DNA (LRV)	Aggregates ^2^ (%)
1	81.8	94.2	87.9	0.4	1.0	1.4
2	86.4	99.5	87.2	0.3	1.1	1.5
3	82.1	94.6	87.6	0.3	1.1	1.4
4	83.7	96.5	87.9	0.4	1.2	1.4
5	85.0	97.9	87.7	0.4	1.1	1.1

^1^ Calculated by the mass of the eluted protein divided by the volume of stacked membranes. ^2^ Mass percentage of aggregates in the feed solution was 3.5% measured according to [55].

## Data Availability

The data presented in this study are available on request from the corresponding author.

## Supplementary Materials

The following are available online at https://www.mdpi.com/article/10.3390/membranes11070530/s1, Figure S1: ATR-FTIR spectra of pristine PBT membrane (PBT), polyGMA grafted membrane (PBT+GMA), and CIX-IDA membrane (PBT+GMA+IDA); Figure S2: Superficial flow velocity versus pressure drop; Figure S3: Chromatogram of five consecutive IgG bind and elute cycles at different RTs from 0.1 to 5.0 min; Figure S4: Equilibrium adsorption isotherm of IgG onto CEX-IDA membranes; Figure S5: Chromatogram of DBC_10%_ measurement at 0.1 min RT; Figure S6: Chromatogram of DBC_10%_ measurement at 1.0 min residence time; Figure S7: Protein G column analysis of protein samples collected from the first cycle of mAb capture from cell culture fluid and S8: Protein G column analysis of protein samples collected from the second to fifth cycles for mAb capturing. Reference [56] is cited in Supplementary Materials.
