# Supplementary material for: Iminodiacetic Acid (IDA) Cation-Exchange Nonwoven Membranes for Efficient Capture of Antibodies and Antibody Fragments
Source: Membranes (Basel). 2021 Jul 14;11(7):530. doi: 10.3390/membranes11070530 (PMC8305546; doi:10.3390/membranes11070530)
Supplement: Supplementary file 1 [file membranes-11-00530-s001.zip › membranes-1283047-SI.pdf]

membranes

MDPI

Supplementary Materials

Iminodiacetic Acid (IDA) Cation-Exchange Nonwoven Membranes for Efficient Capture of Antibodies and Antibody Fragments

Jinxin Fan [1], Cristiana Boi [1,2,3,*], Solomon Mengistu Lemma [2], Joseph Lavoie [1,2] and Ruben G. Carbonell [1,2,4]

[1] Department of Chemical and Biomolecular Engineering, North Carolina State University, Raleigh, NC 27695-7905, USA; jfan22@ncsu.edu (J.F.); jhlavoie@ncsu.edu (J.L.); rgcarbon@ncsu.edu (R.G.C.)
[2] Golden LEAF Biomanufacturing Training and Education Center (BTEC), North Carolina State University, Raleigh, NC 27695-7905, USA; smlemma@ncsu.edu
[3] DICAM, Alma Mater Studiorum-Università di Bologna, 40131 Bologna, Italy; cristiana.boi@unibo.it
[4] National Institute for Innovation in Manufacturing Biopharmaceuticals (NIIMBL), Newark, DE 19711, USA
* Correspondence: cristiana.boi@unibo.it

Citation: Fan, J.; Boi, C.; Lemma, S.M.; Lavoie, J.; Carbonell, R.G. Iminodiacetic Acid (IDA) Cation-Exchange Nonwoven Membranes for Efficient Capture of Antibodies and Antibody Fragments. *Membranes* **2021**, *11*, 530. https://doi.org/10.3390/membranes11070530

Academic Editor: Scott M. Husson

Received: 17 June 2021
Accepted: 11 July 2021
Published: 14 July 2021

Publisher's Note: MDPI stays neutral with regard to jurisdictional claims in published maps and institutional affiliations.

Copyright: © 2021 by the authors. Licensee MDPI, Basel, Switzerland. This article is an open access article distributed under the terms and conditions of the Creative Commons Attribution (CC BY) license (http://creativecommons.org/licenses/by/4.0/).

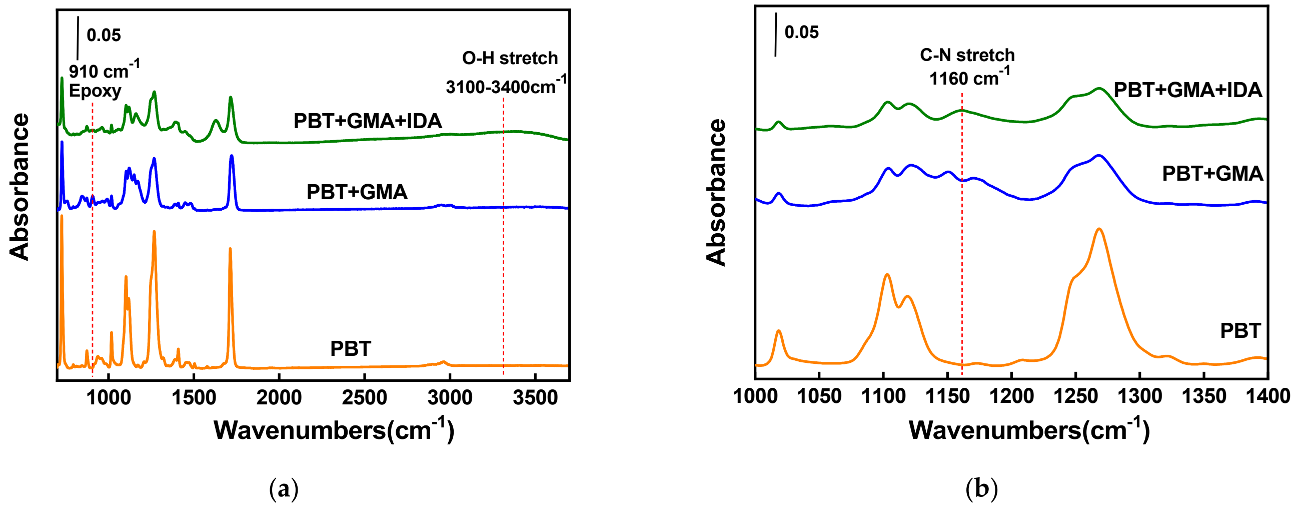

Figure S1. ATR-FTIR spectra of pristine PBT membrane (PBT), polyGMA grafted membrane (PBT+GMA), and CIX-IDA membrane (PBT+GMA+IDA), (**a**) wavenumbers: 700–3700 cm^{-1} and (**b**)wavenumbers: 1000–1400 cm^{-1}.

Pressure Drop Measurements

75 layers of CIX-IDA nonwoven membranes (diameter: 10 mm, area: 0.7854 cm^2) were packed in an Omnifit column holder (diameter: 10 mm) and connected to AKTA system. The pressure drops were measured under a range of superficial velocities from 0-0.34 cm/s with pure water. The pressure drop was calculated by subtracting the pressure drop of empty column from that with membranes in the column. Darcy's law listed as follows was used to analyze the permeability of the membrane packed columns κ.

$$u_0 = \frac{\kappa \times p}{\mu \times L} \tag{1}$$

Here u_0 is the superficial velocity (cm/s); κ is the flow permeability (cm^2); p is the pressure drop (Pa); μ is dynamic viscosity (Pa·s); L is the bed height of the packed membranes (cm). The results are shown in Figure S2.

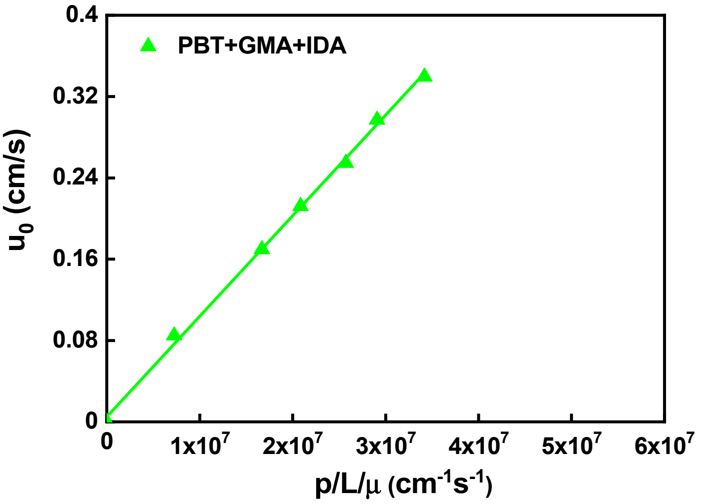

Figure S2. Superficial flow velocity versus pressure drop conducted in a column packed with 75 layers of CIX-IDA membrane (PBT+GMA+IDA), bed height = 1.78 cm. Preparation conditions: polyGMA grafted at 20% WG, IDA coupling for 16 h at 60 °C.

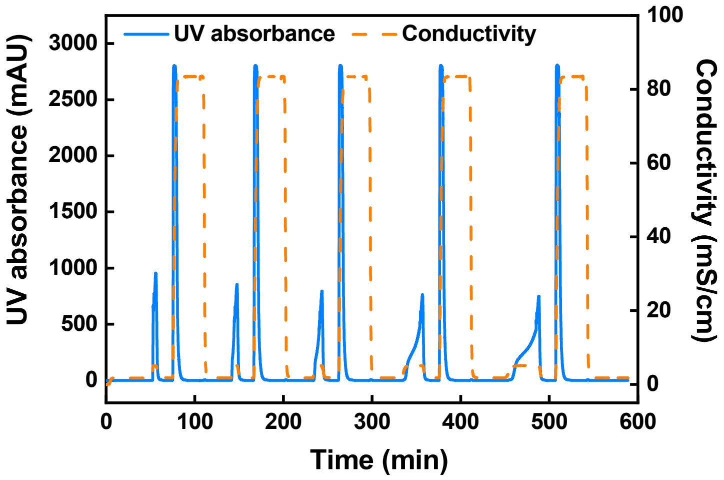

Figure S3. Chromatogram of five consecutive IgG bind and elute cycles at different RTs from 0.1 to 5.0 min. 12 layers with 25 mm diameter of CIX-IDA membranes. Preparation conditions: polyGMA grafted at 20% WG, IDA coupling for 16 h at 60 °C.

Equilibrium Adsorption Isotherm

The IgG adsorbed on the membrane was calculated by dividing the eluted IgG mass by the membrane weight. A range of IgG initial concentrations from 0.2 to 10 mg/mL was employed for binding experiments. The membrane (~10 mg) was incubated in 3 mL of protein solution for 16 h. The Langmuir isotherms listed as follows were used to fit the experimental data [1,2].

$$q_{eq} = \frac{c_{eq} q_m}{c_{eq} + K_d} \quad (2)$$

Here q_{eq} is the amount of IgG adsorbed on the membrane at equilibrium with the IgG in the liquid phase of concentration c_{eq}. The Langmuir parameters are q_m, the maximum binding capacity and the dissociation constant K_d.

After data fitting, the calculated q_m is 648.1 mg/g and K_d is 1.2 mL/mg. The SBC obtained with 10 mg/mL initial IgG concentration was 605.2 ± 19.3 mg/mL where around 8.0 mg/mL IgG remained in the solution after adsorption (shown in Figure S4), and the binding capacity was close to the q_m from Langmuir modeling.

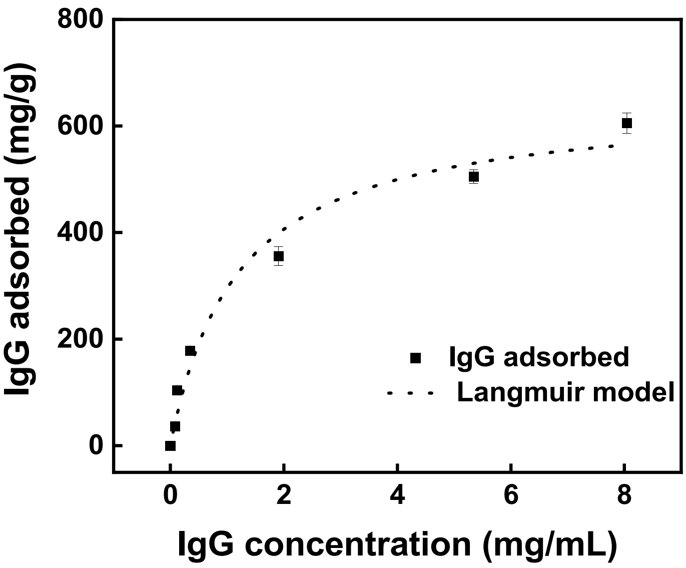

Figure S4. Equilibrium adsorption isotherm of IgG onto CEX-IDA membranes. Experimental data are compared with the Langmuir model. Preparation conditions: polyGMA grafted at 20% WG, IDA coupling for 7 h at 80 °C.

$DBC_{10\%}$ Measurements

A stack of 12 membrane layers (~1.5 mL, 25 mm diameter) was connected on the AKTA system with an Omnifit column holder (diameter: 25 mm). 50 mM acetate buffer (pH 5.5) was used as binding buffer to equilibrate the membranes at 1 mL/min, then 3 mg IgG/mL as the sample solution was loaded with 0.1 or 1.0 min residence time. Loading stopped once UV absorbance reached the value corresponding to 10% of initial feed concentration. After that, the unbound protein was washed away till the UV signal reached the baseline. Finally, the binding buffer with additional 1 M NaCl was used to strip the bound protein (at 1 mL/min). 10% *DBC* was calculated as the following equation:

$$DBC_{10\%}=\frac{C_0\times(V_{10\%}-V_0)}{V_m} \tag{3}$$

Here C_0 is the initial IgG concentration in the feed; $V_{10\%}$ is the effluent volume when the IgG concentration in the effluent reached 10% of the initial IgG concentration in feed (mL); V_0 is the void volume (mL) determined by injecting 1% acetone; V_m is the membrane volume.

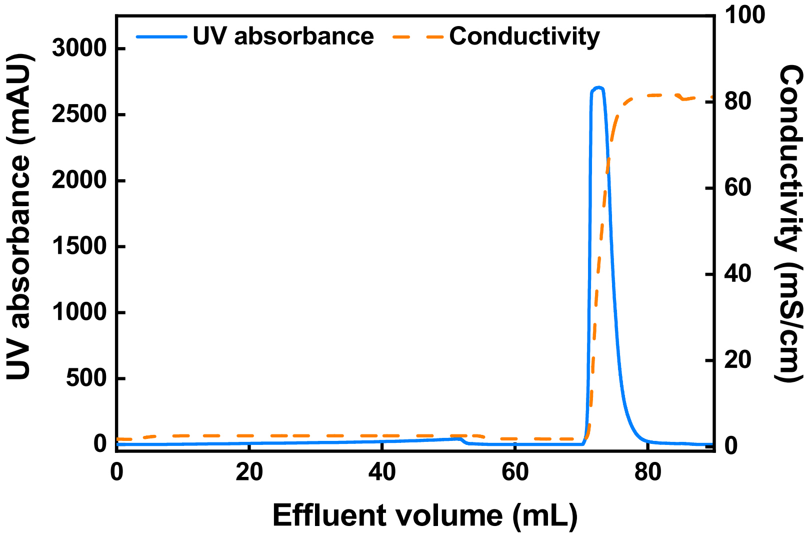

Figure S5. Chromatogram of $DBC_{10\%}$ measurement at 0.1 min RT. 12 layers of CIX-IDA membranes with 25 mm diameter. Preparation conditions: polyGMA grafted at 20% WG, IDA coupling for 7 h at 80 °C.

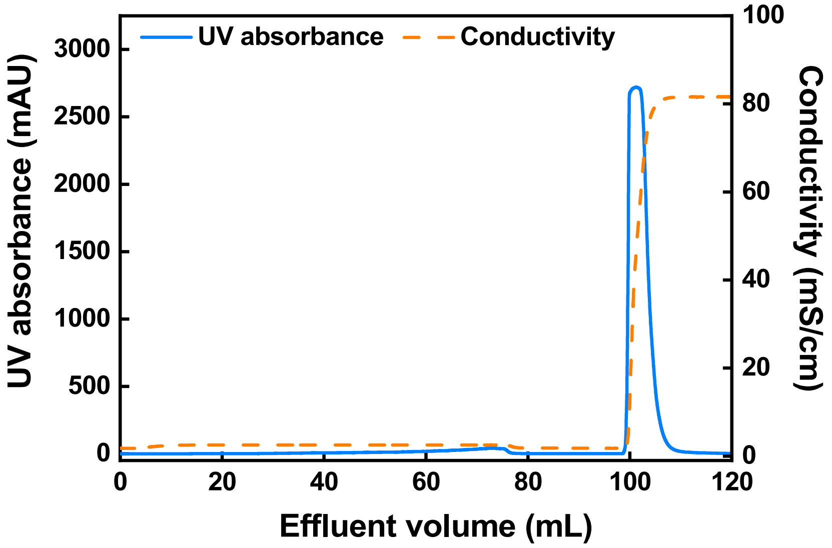

Figure S6. The chromatogram of $DBC_{10\%}$ measurement at 1.0 min residence time (CIX-IDA membrane: the grafted polyGMA WG = 20%, IDA coupling time = 7 h, IDA coupling temperature = 80 °C, 12 layers with 25 mm diameter). Preparation conditions: polyGMA grafted at 20% WG, IDA coupling for 7 h at 80 °C.

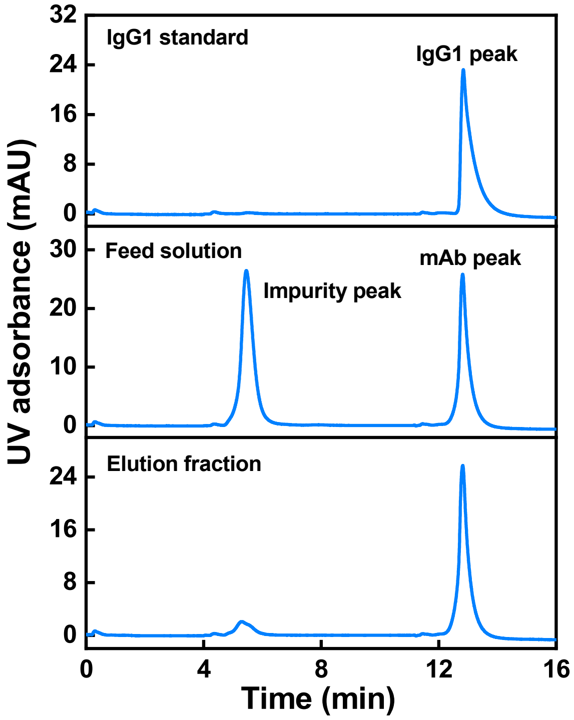

Figure S7. Protein G column analysis of protein samples collected from the first cycle of mAb capture from cell culture fluid (12 layers of CIX-IDA membrane with 10 mm diameter at 1.0 min residence time).

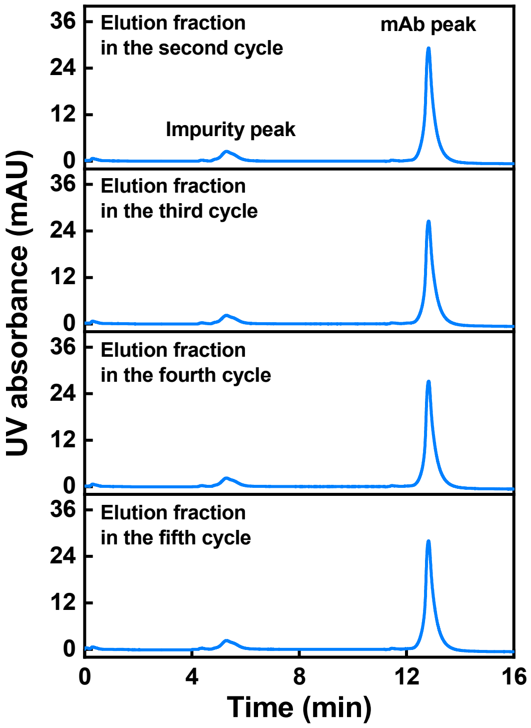

Figure S8. Protein G column analysis of protein samples collected from the second to fifth cycles for mAb capturing.

References

1. Cattoli, F.; Boi, C.; Sorci, M.; Sarti, G. Adsorption of pure recombinant MBP-fusion proteins on amylose affinity membranes. *J. Membr. Sci.* **2006**, *273*, 2–11, doi:10.1016/j.memsci.2005.09.003.
2. Rajesh, S.; Schneiderman, S.; Crandall, C.; Fong, H.; Menkhaus, T.J. Synthesis of Cellulose-graft-Polypropionic Acid Nanofiber Cation-Exchange Membrane Adsorbers for High-Efficiency Separations. *ACS Appl. Mater. Interfaces* **2017**, *9*, 41055–41065, doi:10.1021/acsami.7b13459.
